# Modeling the risk of transmission of schistosomiasis in Akure North Local Government Area of Ondo State, Nigeria using satellite derived environmental data

**DOI:** 10.1371/journal.pntd.0005733

**Published:** 2017-07-12

**Authors:** Oluwaremilekun G. Ajakaye, Oluwatola I. Adedeji, Paul O. Ajayi

**Affiliations:** 1 Department of Crop, Soil & Pest Management, Rufus Giwa Polytechnic, Owo, Ondo State, Nigeria; 2 Department of Strategic Space Applications, National Space Research and Development Agency (NASRDA), Abuja, Nigeria; 3 Department of Geography, Archaeology and Environmental Studies, University of Witwatersrand, Johannesburg, South Africa; University of Florida, UNITED STATES

## Abstract

Schistosomiasis is a parasitic disease and its distribution, in space and time, can be influenced by environmental factors such as rivers, elevation, slope, land surface temperature, land use/cover and rainfall. The aim of this study is to identify the areas with suitable conditions for schistosomiasis transmission on the basis of physical and environmental factors derived from satellite imagery and spatial analysis for Akure North Local Government Area (LGA) of Ondo State. Nigeria. This was done through methodology multicriteria evaluation (MCE) using Saaty’s analytical hierarchy process (AHP). AHP is a multi-criteria decision method that uses hierarchical structures to represent a problem and makes decisions based on priority scales. In this research AHP was used to obtain the mapping weight or importance of each individual schistosomiasis risk factor. For the purpose of identifying areas of schistosomiasis risk, this study focused on temperature, drainage, elevation, rainfall, slope and land use/land cover as the factors controlling schistosomiasis incidence in the study area. It is by reclassifying and overlaying these factors that areas vulnerable to schistosomiasis were identified. The weighted overlay analysis was done after each factor was given the appropriate weight derived through the analytical hierarchical process. The prevalence of urinary schistosomiasis in the study area was also determined by parasitological analysis of urine samples collected through random sampling. The results showed varying risk of schistosomiasis with a larger portion of the area (82%) falling under the high and very high risk category. The study also showed that one community (Oba Ile) had the lowest risk of schistosomiasis while the risk increased in the four remaining communities (Iju, Igoba, Ita Ogbolu and Ogbese). The predictions made by the model correlated strongly with observations from field study. The high risk zones corresponded to known endemic communities. This study revealed that environmental factors can be used in identifying and predicting the transmission of schistosomiasis as well as effective monitoring of disease risk in newly established rural and agricultural communities.

## Author summary

Urogenital schistosomiasis is a parasitic disease whose transmission depends on environmental factors. The disease is one of the risk factors of bladder cancer, the second most common urogenital cancer after prostate cancer in Nigeria. This study aims to identify area suitable for the transmission of the disease through the use of environmental factors. This was achieved by using spatial analysis and multicriteria evaluation for Akure North Local Government Area (LGA) of Ondo State, Nigeria. The results showed that a large portion of the study area was highly favourable for the transmission of the disease. Of the five communities in the study area, only one had a low risk of transmission of the disease while the risk of transmission increased in the remaining four communities. The predictions made by the model was comparable to observations from field study. This study confirmed that analysis of environmental factors using relevant techniques can be applied in the study of transmission of parasitic diseases.

## Introduction

Schistosomiasis is the most important water impounding disease and one of the diseases classified by the World Health Organization (WHO) as neglected. It is the second most prevalent tropical disease in its public health implications. It is widespread in Nigeria and has been estimated that 240 million people required treatment globally [1]. Nigeria has been placed at the top of the list of endemic countries in a more recent estmation [2, 3] as it accounts for 14% of the global number of *Schistosoma* infections. The highest prevalence of infection was found among children of five to ten years of age. Men were more likely than females to have the disease [4]. *S*. *haematobium* is the predominant specie in the country corresponding to 79.8% of all reported cases [5]. It often exceeds the high-endemicity threshold of 50% prevalence. Another recent review points out that there are about 100 new cases of bladder cancer and over 600 deaths annually and the major risk factor is infection with *Schistosoma haematobium* [6]. Thus, the control of schistosomiasis is one of the major prorities of the country’s health system [4].

Urogenital schistosomiasis is caused by the species of flat worms known as *Schistosoma haematobium* [7]. Adults of the parasites live in the blood of mammals but their life cycle requires a phase of asexual multiplication within a fresh water snail host mainly snails of *Bulinus* species. The fluke’s life cycle begins when adult female schistosomes deposit eggs in the veins surrounding the bladder of the mammal host. Eggs are extruded from the veins through the tissues into the bladder and are voided to the exterior in the urine [8]. The eggs that are deposited into fresh water hatch as a result of osmotic stress that causes the shells to rapture and embryos known as miracidia emerge [9]. The ciliated miracidia swim frantically through the water by means of cilia covering the body for several hours until they penetrate a suitable snail. Here, the miracidia undergo further development resulting in the formation of cercariae. The mature carcariae leave at the exit pore located at one extremity and are emitted into the water body by the snail in response to some physico-chemical factors of the water body. The most important of these factors are light and temperature.

Humans acquire an infection through the skin by getting in contact with water containing cercariae shed by the snail hosts. They are attracted to the secretions of the skins showing a strong positive response to arginine [10]. When the larvae come in contact with human skin, they secrete lytic enzyme from postacetabular glands which they use to pierce the skin. The larvae burrow through skin or mucous membrane of a human and once inside shed their forked tail and become schistosomulae. Provided that the human is susceptible to infection, the schistosomulae are carried through the lymphatic system and lungs to the portal venules of the liver. It is primarily in the liver and portal system that larvae develop into adult worms of both sexes [8]. It is also here that they copulate and produce eggs for reproduction of their species [11].

Fresh water snail’s distribution and abundance drive the links between transmission and environment/climate. The distribution of the several *Bulinus* species and possibly the new spread of *B*. *forskalli*, which are two different hosts with two different types of links with the environment, show the impact of environmental factors on the spread of schistosomiasis [12]. This is why temperature, rainfall, proximity to water bodies and other environmental factors may be possible factors and be useful to analyse for risk area determination.

The rapidly changing epidemiology of schistosomiasis necesitates that new approaches are used to study the transmission dynamics of the disease in order to promote prevention and control towards sustainenace of a healthy environmental [13,14]. Of recent, geographic information system/remote sensing (GIS/RS) has become a popular, indispensably available instrument applied in many researches including epidemiology, environmental resource management and programming [15,16,17,14,18,19]. GIS/RS provides an important opportunity of obtaining surface characteristic variables such as humidity, soil temperature and vegetative cover of schistosomiasis endemic areas, fitting these parameters with snail population and or prevalence thereby establishing a credible forecast model of schistosomiasis [13,14,18]. Risk maps are outcomes of transmission models in which environmental information has been merged with data from the fields of epidemiology and vector biology [20]. The potential of the combination of remote sensing and geographical information system based spatial analyses for schistosomiasis risk modeling can be used to determine the geographical limit of disease distribution, understand disease ecology and epidemiology, support prevention, surveillance and control through prioritising areas of disease risk and provide early warning for areas where disease transmission could become established [21].

This study aims at generating a predictive risk model for schistosomiasis at a local level by analysing satellite derived environmental data using different techniques.

## Materials and method

### Ethics statement

Ethical approval was sought and obtained from the Ondo State Ministry of Health, Akure, Ondo State.

### Study area

**Fig 1:**
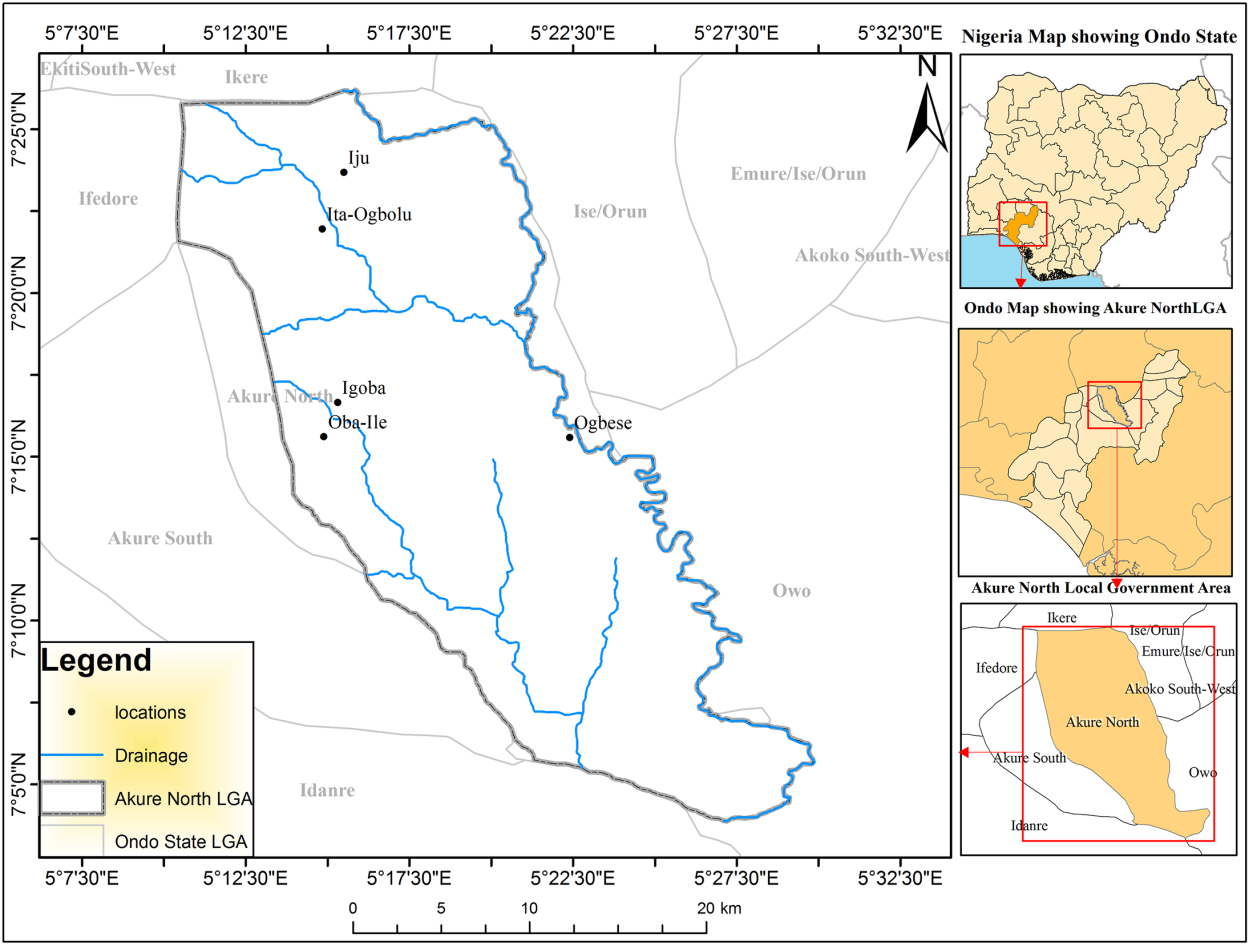
Map of Akure North local government area.

The study area comprises the whole of Akure North Local Government Area in Ondo state of Nigeria. The Local Government Area comprises of five major communities; Iju, Ita Ogbolu, Oba Ile, Igoba and Ogbese in Akure North LGA (Fig 1). These communities are located between latitudes 5°45' and 7°52'N and longitudes 4°20' and6°05'E. The population of the area is approximately 198,000. The vegetation type of the local government area is typically rainforest dominated by abundant trees and grasses. The economic activities in the area includes fishing and production of food and tree crops such as cocoa, rubber, oil—palm, cashew, teak, gmeligna and indigenous tree species. The predominant occupations in the communities are farming and trading. The area has a maze of numerous drainages (Ala, Oluwa, and Ogbese River).

### Determination and standardisation of schistosomiasis risk factors

Every organism has a range of tolerances for every abiotic condition including an optimum range at which it’s most abundant [22]. It has been stated by [23] that temperature is one of the most important physical influences of any biotope from an ecological perspective. Temperature generally affects snail population size and the fecundity of adult snails. Different snail species require a series of optimal temperature tolerance ranges for survival and reproduction [22] but the most favourable range lies between 18°C and 32°C [24]. The influence of temperature on the schistosomes' intra-molluscan stages as a determinant of the parasites' spatial distribution has also been studies by [25]. Eggs of the parasite hatch at temperatures ranging between 10°C and 30°C [26]. The period from penetration of the miracidium to initial shedding of cercariae by the snail varies with temperature between the minimum of 17 days at 30–35°C and several months at lower temperatures. It can be concluded from these studies that thermal variation influences both parasite biology and the rate of natural increase of snail species [27].

Rainfall is an important abiotic condition in the epidemiology of schistosomiasis as it affects the duration of desiccation [28] and the permanence of habitats through droughts and floods. Rainfall also plays a role by changing schistosomiasis transmission foci and affecting the seasonal patterns of cercarial production. The study by [29] on snails concluded that rainfall is one of the most significant factors affecting snail population fluctuations in tropical areas. Rainfall is important to this study because it is an indicator of the availability of water for disease transmission, especially in rain-fed habitats. A seasonal influx of water may also be positively correlated with infection rates and can change the focality of the disease [30]. It also exerts a strong influence on vegetation cover and acts indirectly on the snails through changes in the surrounding habitat's flora and fauna [31].

Elevation has been linked to current velocity. The influence of geomorphology as a significant determinant of current speed in Mpumalanga and Swaziland has been reported by [27] and found that the distribution of hard rock correlates with snail distribution. In a study by [32], an elevational limit of 800m-2200m above sea level was established as suitable elevation for S.mansoni due to hot water temperatures below 800m and cold water temperature above 2200m in Ethiopia. Altitude also influences temperature; the average temperature decrease with height in free atmosphere [33].

Changes in land use such as deforestation, human settlement, and construction of roads, waters dams, canals and irrigation systems have been accompanied by global increases in morbidity and mortality from parasitic diseases. Amongst the factors that determine the nature and extent of change in the incidence of parasitic disease are changes in land use and settlement and the time interval from one land use to another [34]. In China, the construction of the Three Gorges high dam on the Yangtze River affected the snail distribution and annual prevalence of human schistosomiasis varying with water levels in the Yangzte River [35].

Normalized difference vegetation index (NDVI) has been reported to be one of the most successful environmental factors for snail habitat prediction. Normalized Differentiation Vegetation Index (NDVI) represents the amount of chlorophyll in an area. Freshwater snails are usually associated with macrophytes. These leafy aquatic plants provide the host snails with shelter from solar radiation and currents and egg-laying sites [25]. The snails also feed on decaying plants [36]. Also when these plants provide shade, the vegetation moderates the water temperature. The presence of aquatic vegetation is positively linked to the amount of dissolved oxygen and the consumption of carbon dioxide (CO_2_) and thereby linked to movement and reproduction of pulmonate snails [37].

The relationship between the slope and schistosomiasis has been very rarely reported. In a study by [38], it was discovered that slope played an important role in the distribution of *Oncomelania hupensis*, the unique intermediate host snail of *Schistosoma japonicum* in the mouintaneous regions of China.

The location of a household in relation to the suitability of a water body to transmit the disease has shown to be highly relevant with respect to the level of prevalence [39]. A study in Ghana found that high infection levels were clustered around ponds known to contain intermediate host snails of *S*. *haematobium*, whilst prevalence was low in households in close proximity to a non infested river [40]. A study by [41] concluded that the relative location of a house to snail-free or snail-colonised water sources was a key factor explaining the spatial pattern of *S*. *mansoni* infection in Brazil. These risk factors were therefore selected, reclassified and standardised into four levels of risk namely, very low, low, moderate and high risk.

### Satellite data

The datasets used in this research were obtained from different sources based on availability “Table 1”. The thermal band of the Landsat Operational Land Imager (OLI) sensor was used to derive Land surface temperature (LST) over the study area [19]. The digital numbers of band 11 of Landsat OLI was first converted to spectral radiance. Spectral radiance values were then converted to radiant surface temperature. The calculated radiant surface temperatures were subsequently corrected for emissivity and converted into Celsius. The monthly rainfall data was obtained from the European Meteorology Research Programme (www.euramet.org). The Digital Elevation Model (DEM) of the Advanced Space borne Thermal Emission Radiometer (ASTER) was obtained from the National Aeronautical and Space Agency website (www.nasa.gov), the remotely sensed data was captured with a spatial resolution of 30 metres. The Land use data was acquired from the global land-cover website of the University of Maryland, USA (www.landcover.org). NDVI was generated using the near infra red band and the red Band of the Landsat i.e. band 4 and band 3 respectively using the NDVI equation. The equation is explained by subtracting plants reflectance of red light from near–infrared light then dividing the difference by the addition of the red and near infrared light reflected. The slope image was obtained from the Digital Elevation Model and was converted using the spatial analyst tool in the ArcGIS software. Drainages (rivers) of the study areas were obtained from high resolution imagery and buffered from 0m to 2000m using the buffering tool in ArcGIS.

**Table 1:** Characteristics of imagery.

S/N	Source	Date	Data Type	Spatial Resolution
1	National Aeronautical and Space Agency		DEM, Slope	30 meters
2	Landsat Operational Land Imager (OLI). (www.landcover.org)	5th Feb., 2015	Landcover	30 meters
3	Landsat Operational Land Imager (OLI)	5^th^ Dec., 2015	Temperature, NDVI	30 meters
4	European Meteorology Research Programme (www.euramet.org)		Rainfall	0.7metres

### Reclassification

All the prepared raster data layers (criteria) were set to local coordinate system of WGS 1984 at a spatial resolution of 30m. The suitability levels for each of the criterion layers were defined based on literatures, experts’ knowledge and author’s practical experiences [24, 25, 26, 29, 32, 34, 35, 40, 42] except for slope that was based on arbitrary values. The layers were reclassified into different suitability level using reclassify tool in ArcGIS 10.1 as a base to construct the criteria maps.

### Analytical hierarchy process (AHP)

This was done through methodology multicriteria evaluation (MCE) using Saaty’s analytical hierarchy process (AHP). AHP is a multi-criteria decision method that uses hierarchical structures to represent a problem and makes decisions based on priority scales. In this research AHP was used to obtain the mapping weight or importance of each individual schistosomiasis risk factor. The process of deriving the weights of each factor involved the following steps:

1. formulation of a pair wise comparison matrix for each of the six input parameters.
2. establishment of the relative weights of each input parameter.
3. checking for consistency in the pairing process.

The risk factors do not have the same role and weight in the modelling of the final schistosomiasis risk zones. In order to designate the importance of each parameter, they were weighted using a pair wise comparison method which is one of the components of AHP. To assist in the weighting process of the pair wise matrix, the Saaty’s pair wise comparison table “Table 2” was used in the research.

**Table 2:** Saaty’s pairwise comparison table.

Intensity of Importance	Definition of Explanation	Explanation
1	Equal importance	Two factors contribute equally to the objective
3	Somewhat more important	Experience and judgement slightly favour one over the other
5	Much more important	Experience and judgement strongly favour one over the other
7	Very much more important	Experience and judgement very strongly favour one over the other
9	Absolutely more important	The evidence favouring one over the other is of the highest possible validity
2,4,6,8	Intermediate values	When compromise is needed

After computing the pair wise matrix, a measure of consistency (CI) was used to check if the matrix was derived at an acceptable level of consistency using the formula below:

Cl=ƛmax-n/n-1 (1)

Where n is the dimension of comparison matrix, λmax is the maximum eigenvalue of the comparison matrix. The consistency of a pairwise matrix is interpreted as shown in “Table 3”.

**Table 3:** Consistency index interpretation.

Consistency Index	Interpretation
0	*Judgments are perfectly consistent*
≤	Consistent enough
≥	Matrix needs improvement
≥	Judgments are just about random and are completely untrustworthy

The weight values of selected parameters calculated in Analytic Hierarchy Process were used in Weighted Overlay Analysis in ArcGIS to generate the schistosomiasis risk zones.

### Field survey

Buildings were extracted from high resolution imagery for the study through vectorization. The buildings were geocoded i.e. assigned attributes and one out of every ten buildings was selected for urine collection. The map of the study area was printed for field workers and urine was collected from willing participants in each geocoded building.

### Parasitological examination

House to house sample collection was preceded by an interactive meeting of the researcher and village heads during which the purpose of the survey was explained. Each participant was given a clean 50cm^3^ wide-mouthed, screw-capped specimen bottles to supply terminal urine produced between 10:00am and 2:00pm. Each bottle was labeled to correspond to the number of the person on a pre-designed epidemiological form for sampled buildings. The samples were preserved on collection by adding 5ml of 10% formalin at the point of collection and carried to the laboratory. In the laboratory, each sample bottle was agitated to suspend the ova evenly in urine after which 10ml of the urine was transferred with a sterile disposable syringe to a centrifuge tube and centrifuged for 5 minutes at 1500rpm. The supernatant was discarded and the sediment was transferred onto a microscope slide. A drop of Lugol’s iodine was added and neatly covered with a cover slip. The slide was examined under the microscope for eggs of *S*. *haematobium*. When present, the individual was classified as positive for schistosomiasis.

### Statistical analysis

Chi-square statistical analysis was used to test for significant difference between communities and prevalence of infection.

## Results

The reclassification and standardisation of the risk factors is shown in “Table 4”. The suitability levels was represented with intergers and ranked from Very Low to Very high.

**Table 4:** Environmental variables and suitability classes.

	Very Low(1)	Low(2)	High(3)	Very High(4)
LST ^o^C	< 20.0	20.0–22.9	23.0–26.0	> 26.0
Rainfall (mm)	< 1600	1600–1700	1701–1800	>1800
Elevation (m)	> 600	401–600	200–400	< 200
Land Use	Bareground	Settlement	Vegetation	Waterbody
NDVI	No Vegetation	Sparse Vegetation	Less denseVegetation	Dense Vegetation
Slope %	> 30	21–30	11–20	< 10
Proximity (m)	1001–2000	501–1000	101–500	< 100

**Fig 2:**
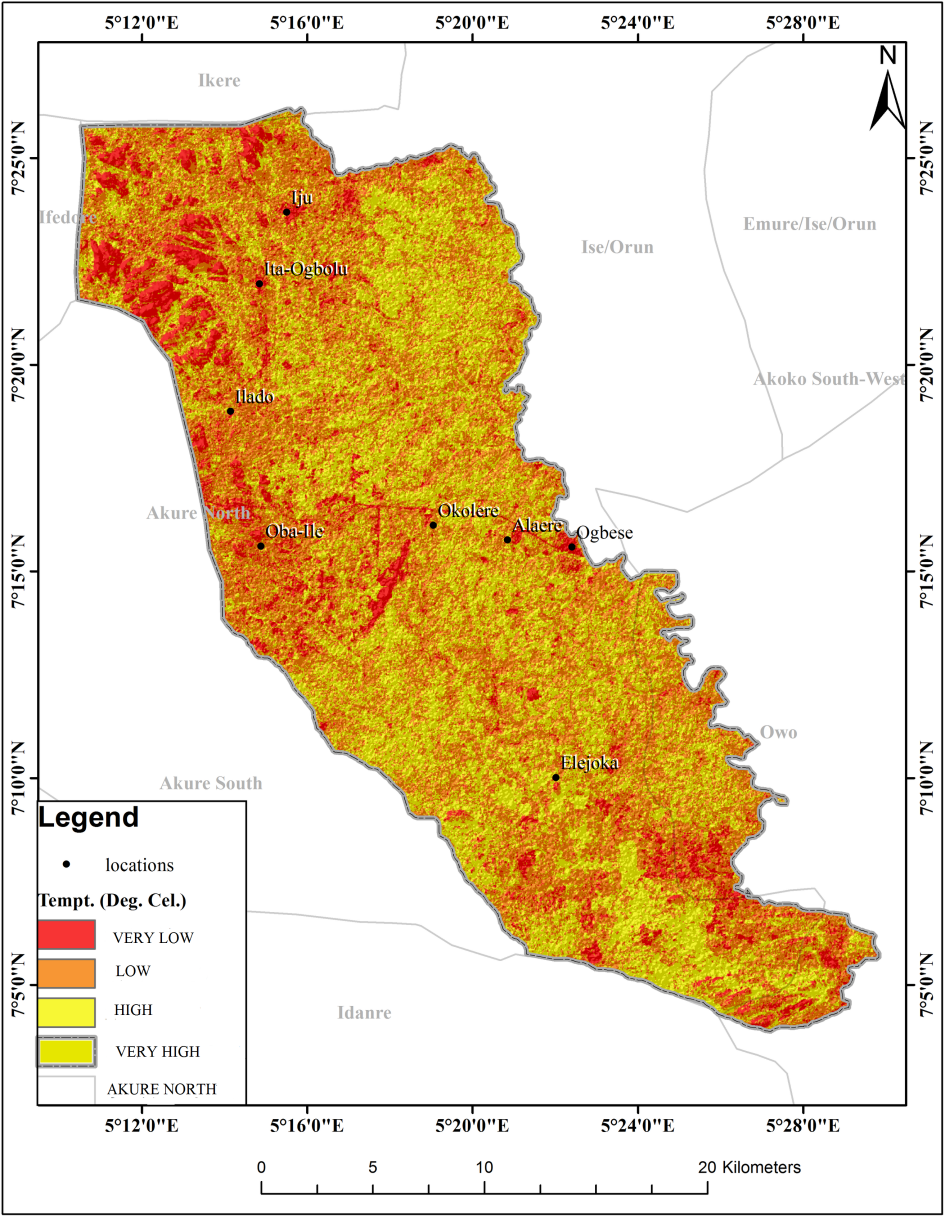
Extraction of environmental data (LST) from remotely sensed images for Akure North LGA.

**Fig 3:**
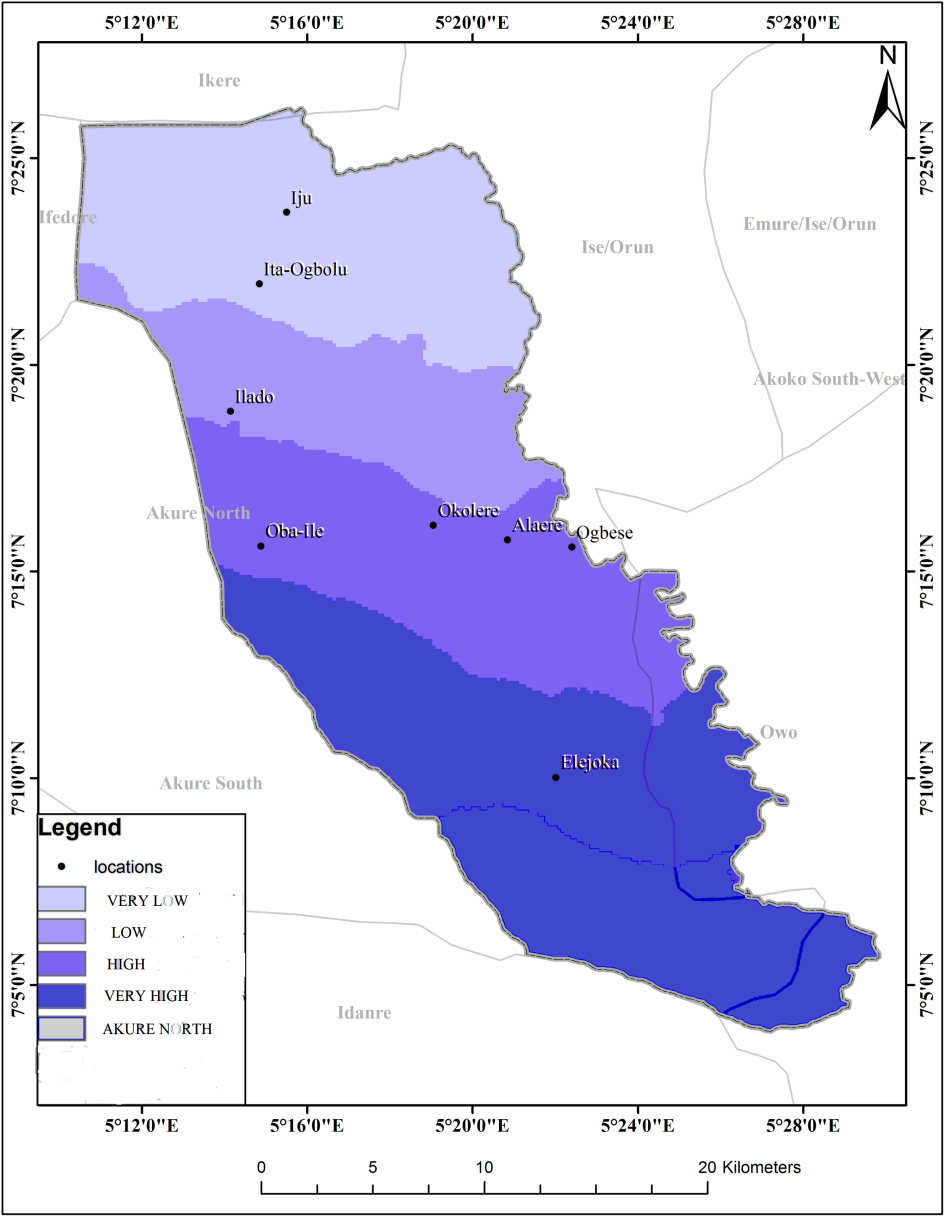
Extraction of environmental data (rainfall) from remotely sensed images for Akure North LGA.

**Fig 4:**
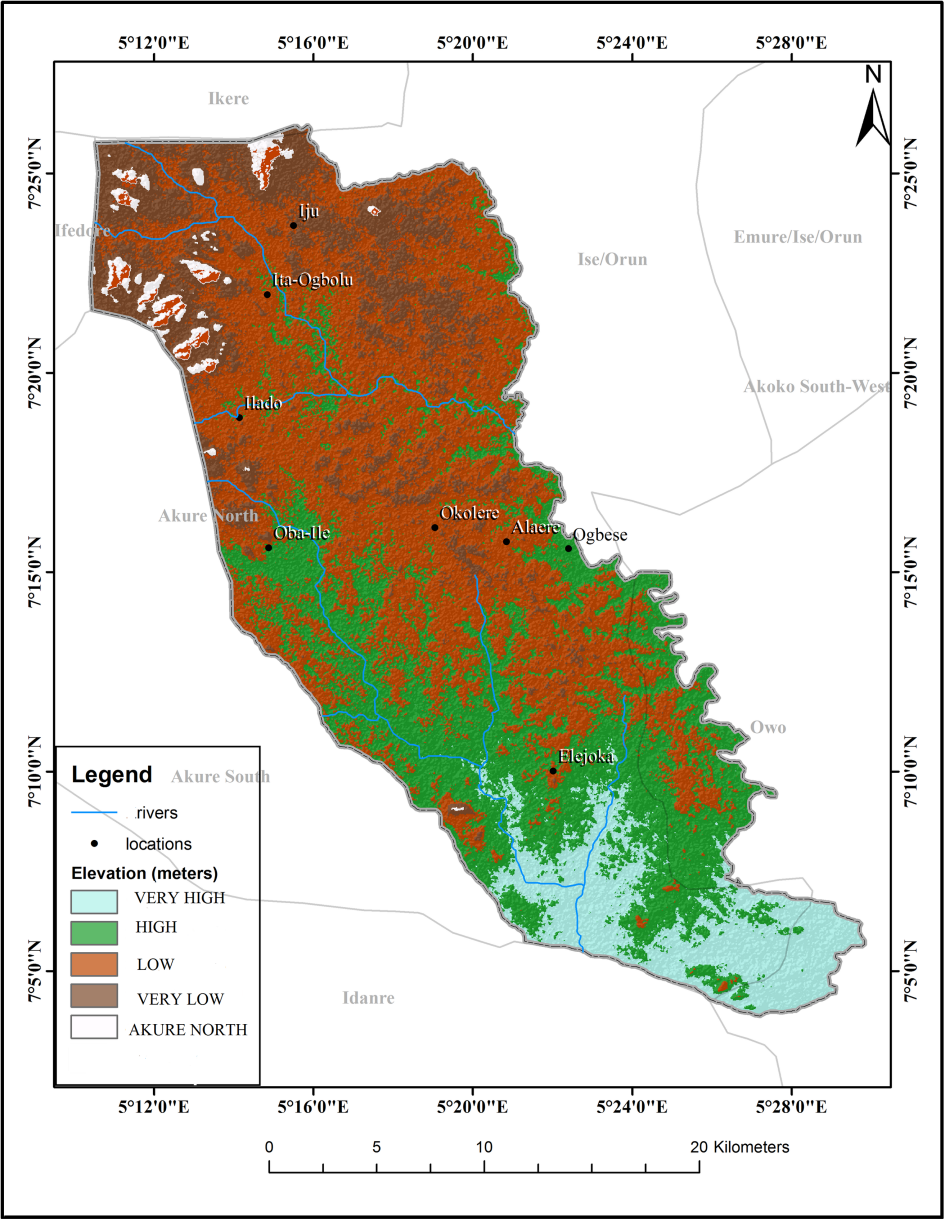
Extraction of environmental data (elevation) from remotely sensed images for Akure North LGA.

**Fig 5:**
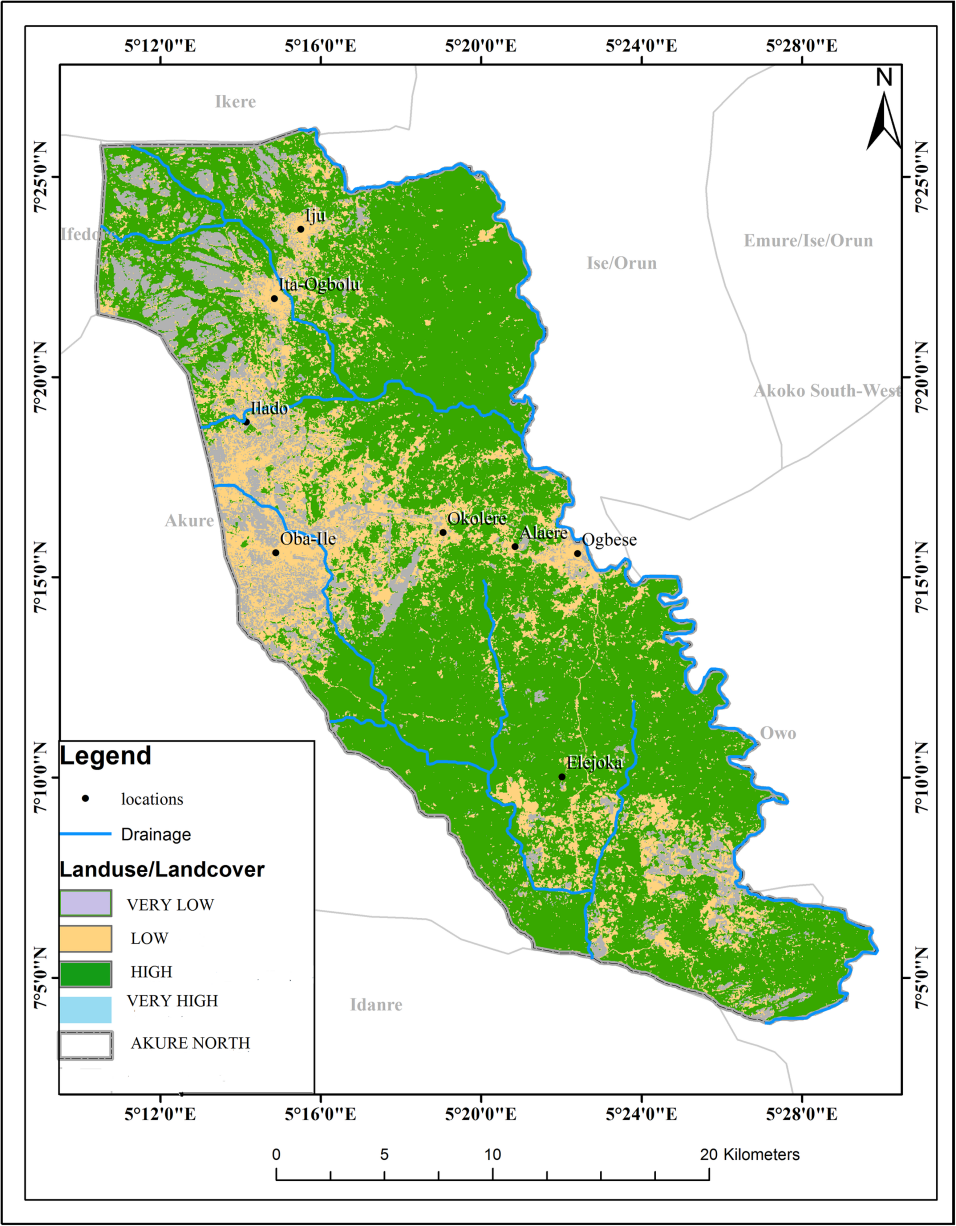
Extraction of environmental data (land use) from remotely sensed images for Akure North LGA.

**Fig 6:**
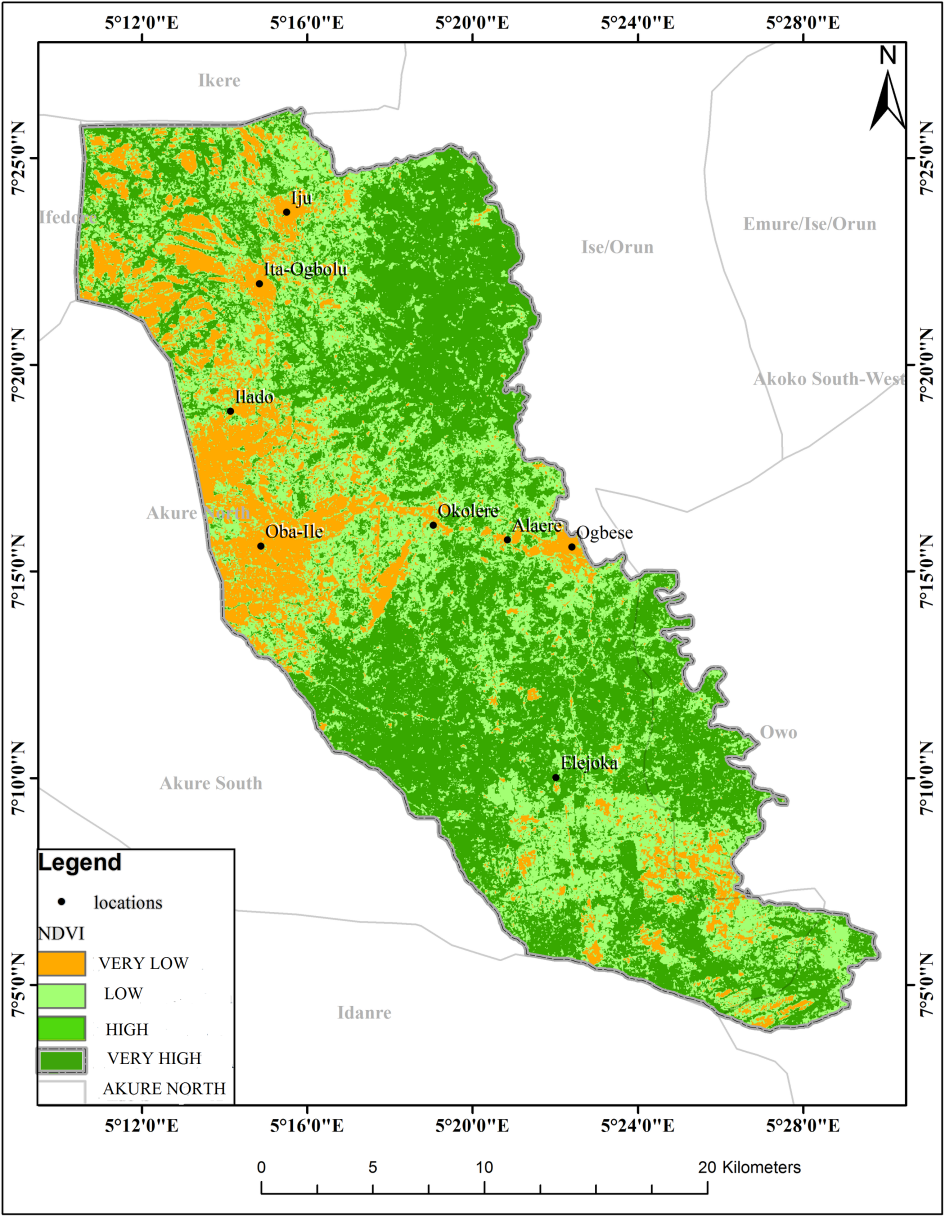
Extraction of environmental data (NDVI) from remotely sensed images for Akure North LGA.

**Fig 7:**
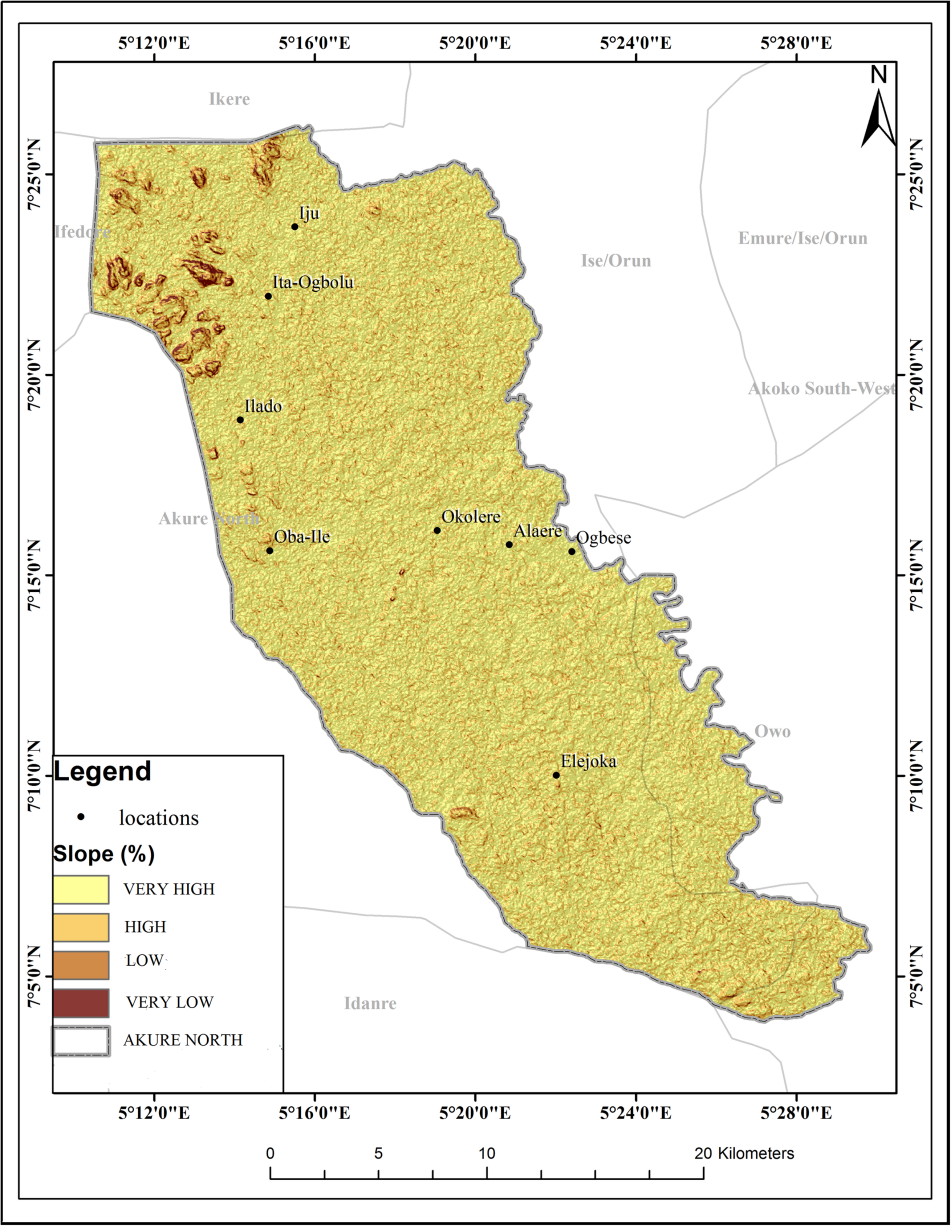
Extraction of environmental data (slope) from remotely sensed images for Akure North LGA.

**Fig 8:**
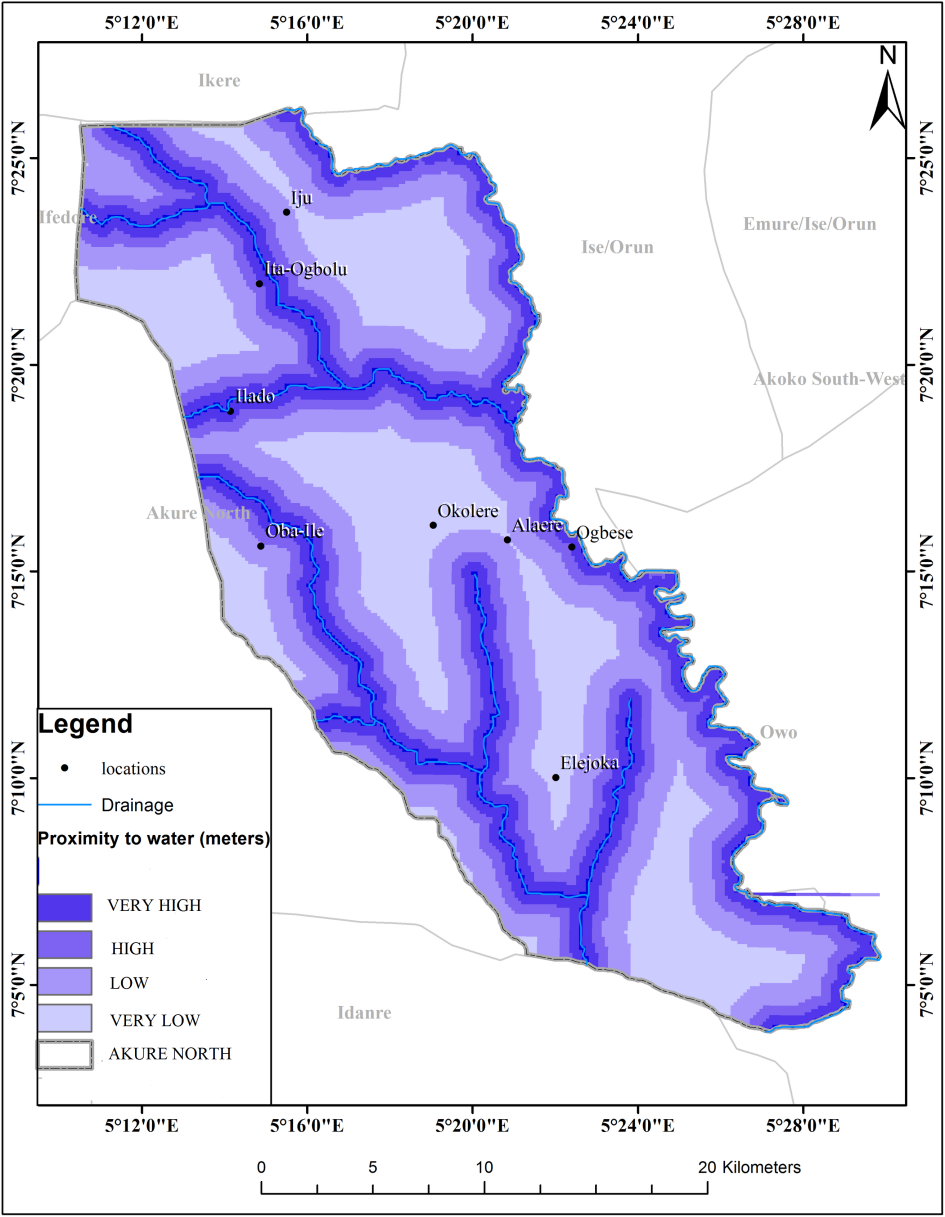
Map showing buffered rivers in Akure North LGA.

The schistosomiasis transmission risk associated with each of the factors that were considered in this research is shown in “Figs 2–8”. “Table 5“ shows the result of the comparison matrix of schistosomiasis risk factors used in the study.

**Table 5:** Comparison matrix of Risk factors used in the study.

	LST	RAINFALL	ELEVATION	LAND USE	NDVI	SLOPE	PROXMITY
LST	1	1.00	5.00	3.00	5.00	9.00	7.00
RAINFALL	1.00	1	3.00	5.00	5.00	7.00	5.00
ELEVATION	0.20	0.33	1	1.00	1.00	5.00	1.00
LAND USE	0.33	0.20	1.00	1	2.00	7.00	1.00
NDVI	0.20	0.20	1.00	0.50	1	6.00	0.50
SLOPE	0.11	0.14	0.20	0.14	0.17	1	0.17
PROXMITY	0.14	0.20	1.00	1.00	2.00	6.00	1

CR = 0.052

The spatial model used to produce schistosomiasis risk zones from risk factors is (Temperature*33.5) + (Rianfall*31.0) + (Elevation*8.2) + (Land use*9.8) + (NDVI*6.7) + (Slope*2.1) + (Proximity*8.6).

**Fig 9:**
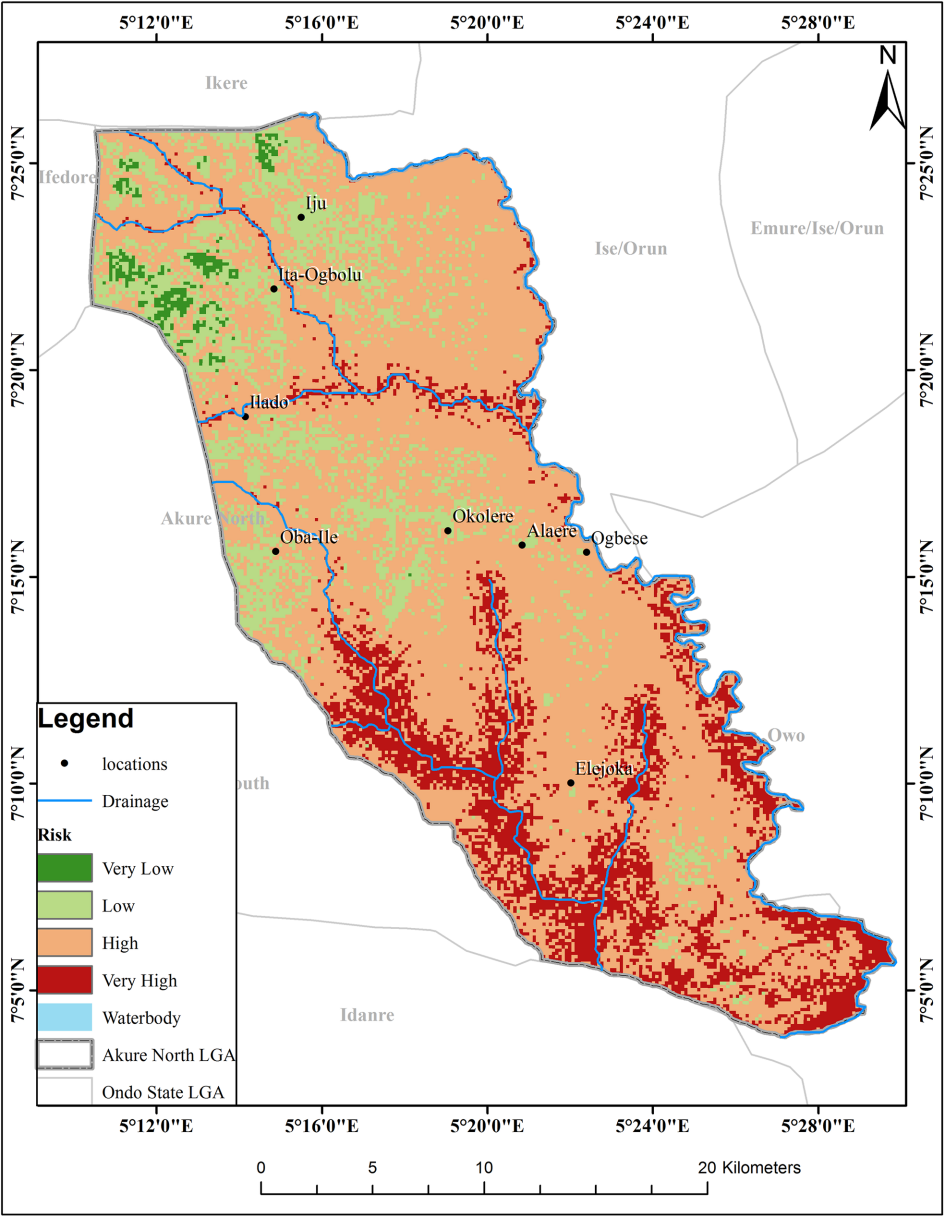
Risk map of schistosomiasis for Akure North LGA.

The map resulting from this weighted sum overlay for schistosomiaisis risk zones is shown in “Fig 9”. The final risk map is based on the best outcome in context of the frequency and transmission of schistosomiasis and classifies the study area into four suitability risk classes. About 110.15km^2^ (15%) of the total study area is subjected to very high schistosomiasis risk, 432.19 km^2^ (57%) labeled as high schistosomiasis risk, and the remaining 98.17 km^2^ (26%) and 7.52 (2%) areas have low and very low schsitosomiasis risk level respectively. Thus, it is possible to conclude that more than 82% of the area with 17,541 buildings is under high risk of schistosomiasis “Table 6”. The map also shows that the northern parts of the region have the lowest risk of schistomiasis while the risk increases as one moves to the southern parts.

**Table 6:** Area covered and number of buildings at risk of in Akure North LGA.

Levels of risk	Area (km^2^)	Number of buildings
Very Low	7.52	0
Low	98.17	8624
High	432.19	17541
Very high	110.15	8917
**Total**		**35,082**

Out of the 1,574 individuals examined in this study, 324 (20.60%) were infected. Infection by the parasite was recorded in all the communities surveyed in this LGA. The highest prevalence of 34.80% was recorded in Ita Ogbolu community while Oba Ile community had the least infection rate of 4.60%. Prevalence of infection differed significantly between these communities (P < 0.05) “Table 7”. This observation is important in future studies on schistosomiasis aimed at studying schistosomiasis transmission and risk in new settlements.

**Table 7:** Prevalence of *S*. *haematobium* infection in Akure North LGA.

Community	No examined	No (%)infected
Igoba	352	57 (16.20)
Iju	265	46 (17.40)
Ita Ogbolu	374	130 (34.80)
Oba Ile	174	8 (4.60)
Ogbese	409	83 (20.30)
Total	1,574	324 (20.60)

## Discussion

The schistosomiasis risk map produced by the study is important as it was generated at a micro scale to show variations in schistosomiasis transmission which may not be noticeable in country wide risk maps. It is also based on environmental conditions, geomorphologic data as well as human induced variables such as landuse.

The existence of suitable climatic conditions, lower elevation, proximities to river, vegetation, high temperatures and rainfall plays a great role for the spread of schistosomiasis in the study area. During the course of the study, it was determined that temperature, elevation and NDVI had the strongest link with the risk of schistosomiasis spread. This corroborate previous researches done in Nigeria and in other parts of the world with the conclusion that environmental factors govern the transmission dynamics of schistosomiasis. In the present study, the high risk areas corresponds to areas with temperature >26^°^C while low risk of infection were found in areas with temperatures <20^0^c. The optimal temperature for snail development and survival is around 25^°^C [43]. The model also suggests that rainfall cannot be used to predict schistosomiasis transmission in the study area. According to [44], the spatial relationship between rainfall and snail population dynamics and infection transmission is difficult to measure since the effect of rainfall varies depending on species of snail and the geographical location. The strong correlation between the risk of schistosomiasis and elevation agrees with investigations in Tanzania and Egypt where altitude was recognized as an important environmental factor in the prevalence of urinary schistosomiasis [15,45]. Suitability was unaffected by slope in this study even though studies on snails host vectors of schistosomiasis showed that snails prefer a slope of less than 20% [46]. The Normalized Difference Vegetation Index (NDVI) is a major environmental factor that can be used in predicting schistosomiasis in the area. In Tanzania and Egypt, NDVI was reported to be a significant environmental variable in schistosomiasis prediction [15,45]. The comparison between risk and proximity to water body in the study under review shows a strong correlation. The high risk zones were located at close proximity to drainages in the study areas. The increase in prevalence with increasing closeness to water might be due to a corresponding increase in water contact activities for domestic, economic or recreational purposes.This has also been observed in study on schools located near Lake Victoria, Kenya where a positive association existed between prevalence of intestinal schistosomiasis and proximity to the lake shores [19].

The field survey records of schistosomiasis prevalence confirm a similar pattern as the spatial epidemiology of schistosomiasis in the region “Table 7”. The results of the Chi square analysis shows that there is significant difference between the communities and prevalence of schistosomiasis.

This research confirmed that satellite derived environmental data can be used to determine schistosomiasis risk levels in an area using relevant techniques. Predictive risk maps can be generated for different areas with different climatic conditions and combined to form country- wide or regional maps. In this study GIS played a good role in the generation of maps for environmental factors, reclassification, overlaying and identification of risk level.

## Conclusion

The model has provided baseline data for schistosomiasis in the study area. It has also highlighted areas of high transmission of schistosomiasis. This is useful in planning and monitoring of control interventions. The results from the field study compared fairly well with the model. The model can be greatly improved by including other significant variables such as snail density in the analysis. The model can also be used to assess control interventions and treatment programs.
